# Hospital Regimens Including Probiotics Guide the Individual Development of the Gut Microbiome of Very Low Birth Weight Infants in the First Two Weeks of Life

**DOI:** 10.3390/nu12051256

**Published:** 2020-04-28

**Authors:** Stefan Kurath-Koller, Charlotte Neumann, Christine Moissl-Eichinger, Raimund Kraschl, Claudia Kanduth, Barbara Hopfer, Manuela-Raluca Pausan, Berndt Urlesberger, Bernhard Resch

**Affiliations:** 1 Division of Neonatology, Department of Pediatrics, Medical University of Graz, Austria Auenbruggerplatz 34/2, 8036 Graz, Austria; 2 Division of Pediatric Cardiology, Department of Pediatrics, Medical University of Graz, 8036 Graz, Austria; 3 Diagnostic & Research Institute of Hygiene, Microbiology and Environmental Medicine, Medical University Graz, 8010 Graz, Austria; 4 Biotechmed Graz, 8010 Graz, Austria; 5 Department of Pediatrics, General Hospital Klagenfurt am Wörthersee, 9020 Klagenfurt, Austria; 6 Department of Pediatrics, General Hospital Hochsteiermark, 8700 Leoben, Austria; 7 Research Unit for Neonatal Infectious Diseases and Epidemiology, Medical University of Graz, 8036 Graz, Austria

**Keywords:** fecal microbiome, probiotics, preterm infant, prevention, necrotizing enterocolitis

## Abstract

Background: It is unknown to what extent the microbiome of preterm infants is influenced by hospital regimens including the use of different probiotics when it comes to the prevention of necrotizing enterocolitis (NEC). Methods: Prospective controlled multicenter cohort study including very low birth weight infants from three neonatal intensive care units (NICUs) between October 2015 and March 2017. During this time span, stool was sampled every other day during the first two weeks and samples were subjected to amplicon-based microbiome analyses. Out of these, seventeen negative controls were processed (German Registry of Clinical Trials (No.: DRKS00009290)). Results: The groups (3 × 18 infants) showed no statistically significant difference regarding gestational age, birth weight, APGAR scores and oxygen demand. 2029 different taxa were detected, including *Enterococcus* and *Staphylococcus*, as well as the probiotic genera *Lactobacillus* and *Bifidobacterium* predominating. The bacterial load was found to increase earlier on when probiotics were used. Without probiotics administration, *Lactobacillus* and *Bifidobacterium* contributed only marginally to the fecal microbiome. Some infants did not respond to probiotic administration. The samples from all centers participating reached a very similar diversity after two weeks while the microbiome samples from all three centers clustered significantly yet varied from each other. Conclusion: Probiotics proved to be safe and initiated an earlier increase of bacterial load (with marked individual divergences), which might play a crucial role in the prevention of neonatal morbidities. Meconium was found not to be free of bacterial DNA, and oral antibiotics did not influence the fecal microbiome development negatively, and hospital regimes led to a center-specific, distinct cluster formation.

## 1. Introduction

About 11% of all live births worldwide are born prematurely (i.e., before 37 weeks of gestational age (GA)), with incidence rates increasing over the past years [1]. Prematurity may be caused by several factors [2] and necessitates intensive care treatment over many months. Long-term morbidities are common among this patient cohort [3].

Mortality is inversely related to GA with necrotizing enterocolitis (NEC) being the most common life-threatening emergency of the gastrointestinal tract with a prevalence rate of 7%–11% in very low birth weight (VLBW; <1500 g) infants [4,5], and an overall prevalence rate of 1%–5% of infants at neonatal intensive care units (NICUs). Mortality rate associated with NEC is up to 30% and fatality rates are inversely proportional with birth weight and GA. Although NEC is a multifactorial disease—this being primarily associated with intestinal immaturity—the concept of “risk factors” for NEC remains controversial; however, the greatest risk factor is prematurity itself [4,5].

The newborn infants´ microbiome originates mainly from vertical transmission of maternal microbiota during delivery, and probably even before [6,7]. Rapidly maturing throughout the first year of life and becoming more or less permanent by three years of age, the individual intestinal microbiome is regulated interactively by initial colonizing microbiota, genetic factors, intestinal development, dietary factors and environmental factors [8,9,10,11].

In the gut of preterm infants, microbial diversity is reduced showing a preponderance of pathogenic organisms [12,13]. Preterm infants carry concurrent immunologic impairment, low intestinal microbiota diversity and predominant intestinal pathogens, and therefore are prone to dysbiosis [9,14,15]. Microbial dysbiosis preceding NEC in preterm infants is characterized both by increased relative abundance of Proteobacteria and decreased relative abundance of Firmicutes and Bacteroidetes [16].

Probiotics may be protective by several potential mechanisms including increasing intestinal barrier function and modification of host response to microbial products [17,18,19,20]. It is not yet known to what extent the microbiome is influenced by hospital regimens during the first days of infant life, including the administration of different probiotics for preventing neonatal morbidities including NEC.

Here, we aimed to characterize the differences of intestinal microbiota composition and abundance with regard to different hospital regimens for NEC prophylaxis. Our hypotheses were that (1) the abundance of the probiotic strains would turn out to be dominant within the fecal microbiome profile, and (2) that administering an oral antibiotic would negatively influence diversity or abundance of microbiome signatures.

## 2. Patients and Methods

### 2.1. Study Design, Recruitment and Ethics

We conducted a prospective controlled triple-center cohort study including VLBW infants from three NICUs applying different hospital regimens for NEC prophylaxis. While carrying out the study, Center G (Graz) used a combination of *Lactobacillus rhamnosus*, gentamycin and nystatin. Center K (Klagenfurt) used *Bifidobacterium infantis*, *Lactobacillus acidophilus* and fluconazole. Center L (Leoben) used gentamycin and nystatin but no probiotics. Details on regimens are given in Table 1. 

Inclusion criteria included a birth weight <1500 g, treatment at the local NICUs between October 2015 and March 2017, and survival of the first three weeks of life. Exclusion criteria included birth weight ≥1500 g, death within the first three weeks of life, genetic diseases, syndromes or congenital anomalies as well as meconium ileus. Stool samples were taken from each infant every other day throughout the first two weeks of life, starting with meconium. Enteral nutrition contained pooled or pasteurized breast milk with subsequent transition to the infants’ mothers´ breast milk at centers G and L. At Center K, mainly preterm formula was used. Table 1 depicts the local regimens used. A detailed study protocol has been published recently [21]. The NEC incidence in Southern Austria was found to be 2.9% (Center G: 2.7%, L: 4.6% and K: 2.2%) during the study period of 2007 to 2016. NEC incidence was found to be significantly higher in center L compared to G and K (*p* < 0.05) (Data from Wellmann F. Epidemiology of necrotizing enterocolitis in Southern Austria. Diploma thesis, Medical University of Graz, 2018).

The local ethic committees approved the study protocol (number 27-366 ex14/15). All infants were recruited after receiving parents´ written informed consent. Routine clinical work-up was not changed for study purposes. All measures were carried out according to good clinical practices.

### 2.2. Microbiome Analyses

Total DNA from frozen stool samples was isolated using MagnaPure LC DNA Isolation Kit III (Roche) according to manufacturer’s instructions. Hypervariable regions V4 were amplified with primers 515F and R926 [22] and sequenced using Illumina MiSeq with v3 600 chemistry [23]. PCR was run in triplicates and pooled subsequently. Library preparation and sequencing of the amplicons were carried out at the Core Facility Molecular Biology at the Center for Medical Research at the Medical University Graz, Austria. The number of bacterial 16S rRNA gene copies was determined using a SYBR based qPCR with primer pair Bac331F and RBac797 [24] according to Pausan et al. 2019 [25]. Reagents were pipetted using Hamilton Starlet and run on a CFX384 Touch™ Real-Time PCR Detection System (Bio-Rad). In total, 17 negative controls were processed and taken into account in subsequent analysis. Thirteen negative controls (one per batch a 96 samples) were blank controls and processed like samples. Raw reads are publicly available at the European Nucleotide Archive (ENA) under BioProject No. PRJEB37883.

Reads in negative controls were interpreted either as contamination from external origin (reagent, environment), or potential cross contamination leaking from samples with high DNA content. Therefore, means of reads per Amplicon Sequence Variant (ASV) per batch were calculated and ASVs were deleted if [(reads in batch NC)/(mean sample reads per batch)]*100 > 20. This approach was quality checked with R package decontam [26] yielding highly similar outcomes.

The MiSeq data analysis was performed using QIIME2 [27] as described previously [28]. The obtained feature table and taxonomy file were used for further analysis and are available on request. Alpha diversity indices and Principle Coordinates Analysis (PCoA) plots based on Bray–Curtis dissimilarities as well as bubble plots and violin plots of Shannon indices, richness and evenness were calculated and constructed using Calypso [29] and R [30] using the package ggplot2 [31]. Differences in the alpha diversity indices between the groups were tested using Qiime2 [27] and Adonis analysis in Calypso. Differences in the fecal microbiomes between the three centers were displayed either in alpha- or beta-diversities. Alpha-diversities were calculated for each sample separately and tested for significant differences between the centers. Richness and evenness, contributing to alpha diversity, were observed separately. Beta-diversities between the centers were displayed in PCoA plots based on unweighted Bray–Curtis dissimilarities.

## 3. Results

In the course of our study, we collected a total of fecal samples from 54 premature infants. Table 2 depicts perinatal and neonatal data of the study population.

**Figure 1:**
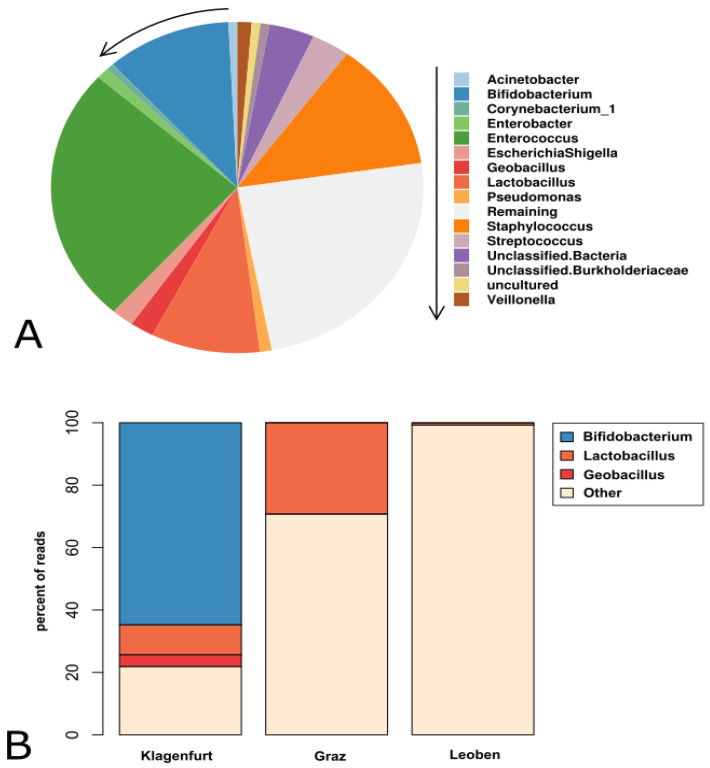
Main bacterial signatures in the premature infants’ gut. (**A**) Pie chart displaying the 15 most abundant microbial genera detected in fecal samples of 54 preterm infants from three centers during the first two weeks of life. (**B**) Relative abundance of signatures from *Bifidobacterium, Lactobacillus, Geobacillus* and other genera in samples from the three centers (K, G and L).

**Figure 2:**
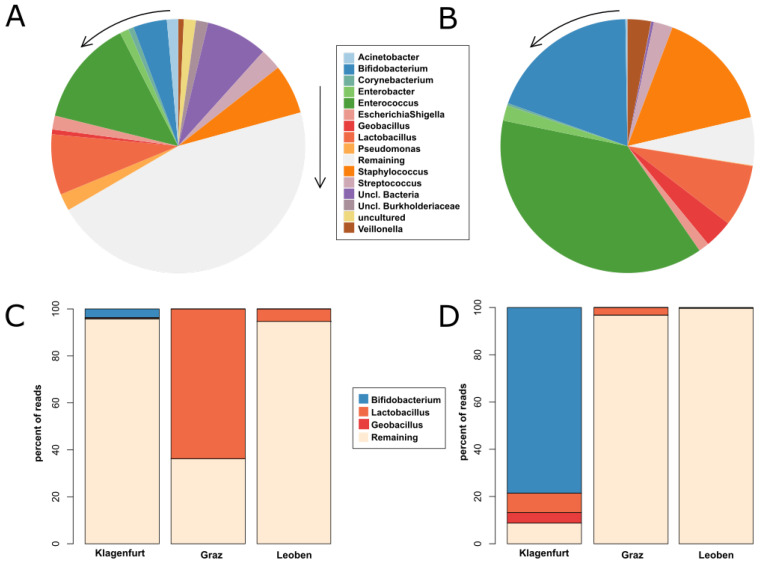
Main bacterial signatures in the premature infants’ gut. (**A**) Pie chart displaying the 15 most abundant microbial genera detected in fecal samples of 54 preterm infants obtained from the three participating centers in the meconium (time points t1 and t2) and (**B**) at time point t6 and t7. (**C**) Relative abundance of signatures from *Bifidobacterium, Lactobacillus, Geobacillus* and remaining genera in samples from the three centers (K, G and L) at time points t1 and t2 as well as (**D**) at time points t6 and t7.

Overall, 383 samples and 16 negative controls, totaling more than 30 million reads, were processed for analysis. After removal of contaminated reads, 2029 different taxa were detected, with *Enterococcus* and *Staphylococcus*, as well as the probiotic genera *Lactobacillus* and *Bifidobacterium* predominating (Figure 1A). Figure 2 depicts the differences between meconium (Figure 2A,C) and the stool at the age of two to three weeks (Figure 2B,D). Meconium was found not to be free from bacterial DNA and probiotics were found to have influenced the fecal preterm microbiome very early on.

Infants’ metadata showed no statistically significant difference regarding GA, sex, BW, APGAR scores and oxygen demand. However, differences were revealed regarding lengths of hospital stay [G: mean 72 days (minimum 25, maximum 126); K: mean 68.5 days (minimum 51, maximum 87); and L: mean 58 days (minimum 24, maximum 92)]. 

**Figure 3:**
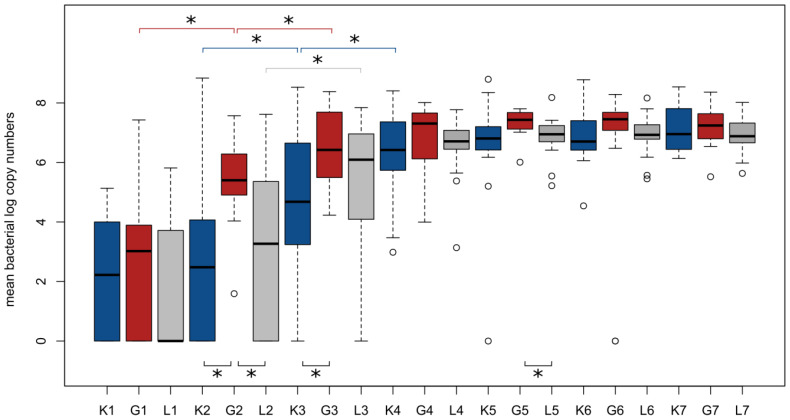
16S rRNA gene copies (mean, standard deviation (SD) and overall variance) per approximately 0.2 mg stool detected via bacteria-specific qPCR in the samples of the three centers K, G, and L, respectively. The last digit indicates the time point. K1 thus refers to t1 in center K. Asterisks (*) indicate significant increases in bacterial load at time points t1, t2, and t3. Outliers are displayed as circles.

The load of microbial 16S rRNA gene copies as obtained by qPCR differed regarding center and time point of sampling (Figure 3). Bacterial load was found to be low in meconium and increased in subsequent samples. In Center G, bacterial load increased earlier compared to centers K and L (Figure 3). After time point t4, the bacterial load remained stable in all samples.

A similar pattern was observed regarding the number of amplicon reads received via next generation sequencing. However, the mean of all read numbers was found to be lower in samples from Center L (no use of probiotics), revealing only 19,338 sequences per sample compared to 37,800 in Center G and 29,401 in Center K (*p* < 0.025). Table 3 depicts the time points of stool sampling.

Microbial signatures were dominated by administered probiotic genera, which is reflected in high absolute and relative abundances in samples from centers G and K (Figure 1B). Almost three quarters of all reads received from Center K were represented by *Bifidobacteria* (64.7%) and lactobacilli (9.6%). In samples obtained from Center G, lactobacilli amounted to 29.2%. In the absence of probiotic administration, the contribution of *Lactobacillus* and *Bifidobacterium* to the microbiome was found to be drastically decreased (Center G: 0.005% *Bifidobacteria*; Center L: 0.677% lactobacilli and 0.088% *Bifidobacteria*). Notably, *Geobacillus stearothermophilus* represented 3.81% of all bacterial reads in Center K, but was not detected in centers G and L.

In Center G, lactobacilli were detected at highest levels early at t2 and t3 (100%) with significant inter-individual variations. Thereafter, absolute and relative abundances decreased (t4 to t5 *p* = 0.03 and *p* = 0.014, respectively). In Center K, peak *Lactobacillus* reads were found at later time points (t5 and t6). However, read numbers were lower compared to Center G. Nearly no *Lactobacillus* signatures were detected in samples from Center L (Figure 1B). The differences between meconium and stool at the end of the second week of life are shown in Figure 2B,D.

At any point in time, signatures of *Bifidobacterium* were more abundant than lactobacilli, although both probiotic strains were administered at the same dosage. *Bifidobacterium* signatures increased over time, displaying highest levels at early time points (t2 and t3, *p* = 0.024). In some samples, up to 97% of reads were derived from the *Bifidobacterium* genus.

Notably, certain infants from centers G (5/18) and K (5/18) did not show any response to probiotic treatment, with restricted colonization by *Lactobacillus*, as compared to the other infants in the same study group (“high responders”). Such a different response was not observed in connection with *Bifidobacterium*.

In Center K, relative abundance of *Geobacillus* signatures increased slightly over time but was overall lower (<4%) compared to *Lactobacillus* or *Bifidobacterium* signatures.

Generally, as the highly abundant probiotic genera *Bifidobacterium*, *Lactobacillus* and *Geobacillus* (BLG) potentially masked the effects of lower abundant taxa, we generated a second dataset, in which we excluded all ASVs affiliated to those genera (dataset “without BLG”). 

After two weeks, samples from all participating centers were found to have reached a similar diversity (t6 and t7). The highest alpha diversities (Shannon Index, reflecting diversity, richness and evenness) were observed in Center K. Differences amongst centers were observed up until t5. Variances in richness and diversity of gut microbiomes between the infants were higher at earliest t1 and decreased over time with and without BLG.

Diversity of the microbiome developed similarly in centers G and L, which both used oral antibiotics. Overall, variances were higher, and richness was lower without BLG.

**Figure 4:**
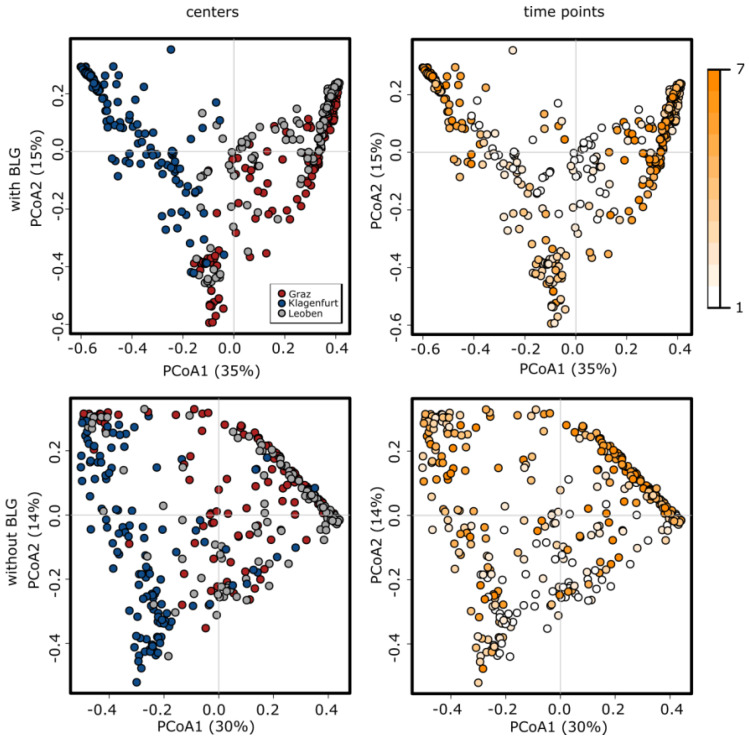
PCoA (Principal Coordinates Analysis) plots displaying the changes in microbial profiles in samples from the three centers over time. Analysis shown with BLG (*Bifidobacterium, Lactobacillus, Geobacillus*) included (upper panels) and without (removed from the dataset, lower panels). Centers are indicated by different colors (red: G, blue: K, grey: L; left panels). Time points of sampling are indicated by shades of orange (white: t1, dark orange: t7; right panels).

**Figure 5:**
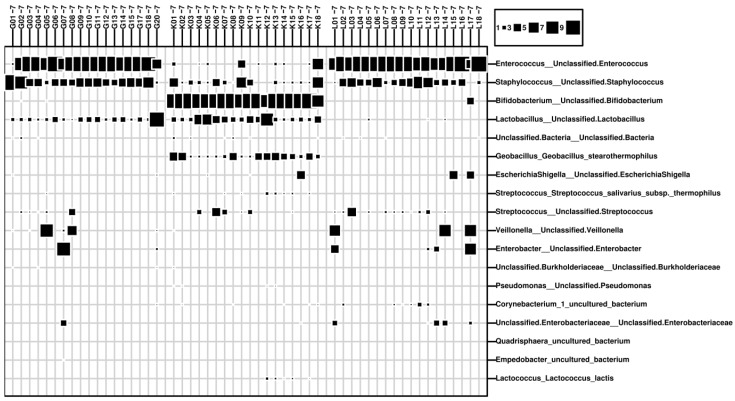
Bubble plot of samples from the three centers at t7 (BLG included), displaying the relative abundance of the 18 most abundant microbial taxa.

During the study we also performed beta diversity analyses based on unweighted Bray–Curtis distances on ASV level to identify overall factors shaping the infants’ microbiome. Using the dataset with BLG included, samples formed center-specific clusters (*p* < 0.001). Center K was found to differ most widely, this being mainly due to probiotic strains (Figure 4). However, even without probiotics (BLG excluded from analysis), clusters were different and center-specific. Notably, cluster formation was found to be time-dependent. Early samples clustered more closely to each other and varied according to the specific center, and the samples dispersed at later time points. However, the inclusion or exclusion of BLG signatures from the analysis did not have an effect on these findings (Figure 4). This indicates that different hospital regimens led to different microbiome profiles and loads over the first two weeks of life not only on the basis of the probiotics used (Figure 5). 

## 4. Discussion

Our results demonstrate that the hospital NEC prophylaxis regimens have a significant impact on the microbiome profile and development in very low birth weight preterm infants.

In this study, bacterial signatures were detectable as early as in the meconium. The microbial load was found to increase earlier when probiotics were administered. In the absence of probiotics, lactobacilli and *Bifidobacteria* contributed only marginally to the fecal microbiome profile. The use of an oral antibiotic for prophylaxis of NEC was found to not be associated with a reduced diversity or reduced abundance of microbiome signatures (disproval of hypothesis 2). Samples from all centers reached a very similar diversity after two weeks, whereas the microbiome compositions were found to be unique, leading to a center-specific, distinct cluster formation.

In the gut microbiome of preterm infants having received probiotics, signatures of probiotic genera were detectable at high numbers (confirmation of hypothesis 1). The fact that in Center K the amount of *Bifidobacterium* reads was higher than the amount of *Lactobacillus* reads although the administered dose was equivalent might be explained by methodological issues (e.g., primer bias) or ecological reasons (e.g., advantage of *Bifidobacterium* in resources competition). Thus, ASV counts only served as estimates.

The strong increase of bacterial load in Center G at early time points t1 to t3 appeared simultaneously with a strong increase of *Lactobacillus* reads in the same samples and thus was most likely caused by the probiotic strain used. No probiotic genera were detected in stool samples when infants did not receive probiotics. Hence, our findings support the current opinion that *Bifidobacterium* and *Lactobacillus* do not act as main contributors to the intestinal microbiome of preterm infants within their first days of life [32].

The impact of probiotics and antibiotics on bacterial load in stool samples needs to be discussed in the light of quantitative PCR data. Fecal bacterial load was found to increase significantly at all three centers during the first week of life, and remained stable thereafter. One would expect oral antibiotics to diminish the bacterial load and probiotics to increase the bacterial load. However, looking at data from Center L, we can see that the administration of oral antibiotics did not result in significantly lower bacterial loads. Even though the bacterial load increased within the first days of life with a certain delay at Center L, levels were comparable between centers until the end of the first week of life. Furthermore, the presence of probiotics in absence of antibiotics did not result in faster increases of bacterial load. In fact, the fastest increase of bacterial load became evident when both probiotics and antibiotics were administered (Center G). Additionally, the role of breast milk feeding in Center G must be taken into account. Individuality was found to be a major driver in the composition of microbiota which was more pronounced in breastfed infants compared to formula-fed infants [33]. Notably, the administration of probiotics or antibiotics had no further impact on the load of bacteria in stool samples of preterm infants after their first days of life.

Besides the probiotic genera, high numbers of *Geobacillus* signatures were found in Center K without there being an intentional administration to the infants. The origin of these bacterial signatures remained speculative. *Geobacillus stearothermophilus* is a thermophilic spore forming bacterium known to spoil food, and especially contaminates whole milk powder [34]. *G. stearothermophilus* is not known as a pathogen nor as being connected to any negative health outcome [34]. It has been detected on surfaces at the NICU [35].

Relative abundances of both probiotic genera have revealed inter-individual differences resulting in great variances at single time points. Individuality has already been described to be a significant factor of microbiome development irrespective of breast milk or formula feeding [33]. Hence, infants seem to respond differently to treatment with probiotic *Lactobacillus* genera. In adults, non-responders to probiotic treatment are well known in the literature [36]. Our data support these findings of individual response to probiotic interventions already in preterm infants [37].

Over time, relative abundances of probiotic genera were found to decrease. Since probiotics dosages for each center remained constant over time in our study, decreasing relative abundances were most likely attributed to the growth behavior of different bacteria within the developing gut. However, any persistence of probiotic genera following a stable colonization could not be assessed in this study as infants’ microbiomes were not analyzed after the course of treatment.

Clustering analyses of samples from the three centers clearly showed that NICUs shape the neonatal intestinal microbiome significantly. Even after exclusion of *Bifidobacterium*, *Lactobacillus* and *Geobacillus*, cluster formations and differentiations were found to remain statistically significant between the three centers. Besides probiotics, environmental factors and medication were also found to contribute substantially to the development of the intestinal microbiome (e.g., age and length of stay at the NICU) 

Notably, *Klebsiella* species were recently claimed as one major factor correlating with NEC in pre-NEC samples [38]. However, in our study, 16 samples from nine infants showed signatures of *Klebsiella*, but none of the infants were diagnosed as having had NEC, and all children bearing *Klebsiella* signatures also remained healthy.

The last samples (at two to three weeks of life) represented the most developed microbiome and maximum display impact of the different NEC prophylaxis regimens. The diversity of the neonatal intestinal microbiome might play a critical role in the long-term sequelae [39]. In this way, our findings could perhaps contribute to a better understanding of the early use of probiotics and oral antibiotics when it comes to the development of microbiomes in preterm infants.

### Limitations

This study was unfortunately limited with respect to the number of recruited patients (18 per center) and the type of microbiome analyses (amplicon-based NGS). Future studies should include metagenomics and metabolomics to allow for conclusions regarding the mechanistic impact from different hospital regimens, as well as to assess the antibiotic resistome and to identify any harmful metabolites. Moreover, the source of the *Geobacillus* signatures in samples from Center K remain unverified for the time being. All of these shortcomings will hopefully be addressed in future studies by including analyses of the administered formula milk. QPCRs specific to *Lactobacillus* and *Bifidobacterium* would further help to estimate the abundance of the probiotic genera more precisely, as could the inclusion of cultivation-based strategies.

## 5. Conclusions

We found that hospital regimens significantly influenced the microbiome composition, diversity and quantity right from the beginning and all the way up to the more mature microbiome starting from two to three weeks of age onwards. Probiotics were found to initiate an earlier increase of bacterial load, and this in turn might play a role in the prevention of neonatal morbidities. However, we have also observed some marked individual divergences. Meconium was not found to be free from bacterial DNA and oral antibiotics seemed to have no negative influence on the fecal preterm microbiome. Our findings might be important for interpretation of microbiome analyses in comparable settings.

## Figures and Tables

**Table 1:** Regimens regarding administration of medication, feeding and probiotic treatment at the three Neonatal Intensive Care Units (NICUs). p.o.: per os (oral); BM: breast milk.

	NICU Graz	NICU Klagenfurt	NICU Leoben
Probiotics	*Lactobacillus rhamnosus* 1 × 10^9^ CFU/d p.o., split into 2 doses per day	*Bifidobacterium infantis* 2 × 10^9^ CFU/d *Lactobacillus acidophilus* 2 × 10^9^ CFU/d in combination, p.o.	None
Antibiotics	Gentamycin 7 mg/kg, every 12 h p.o.	None	Gentamycin 7 mg/kg, every 12 h p.o.
Antifungal agents	Nystatin 10,000 U/kg every 6 h p.o.	Fluconazole 6 mg/kg i.v. every 72 h (<1000 g BW)	Nystatin 10,000 U/kg every 6 h p.o.
Feeding	Pooled or pasteurized BM; subsequent transition to mother´s BM or preterm formula (hydrolyzed if BW <1000 g)	Preterm formula and in few cases additionally pasteurized BM (no pooled BM)	Pooled or pasteurized BM; subsequent transition to mother´s BM or preterm formula (hydrolyzed if BW <1000 g)

**Table 2:** Perinatal and neonatal data of the study population.

	Center G	Center K	Center L	*p*-Value
**Number**	18	18	18	
**Male/female**	10/8	12/6	12/6	0.61
**BW (g)**	1030 (550; 1495)	973 (560; 1485)	1185 (640; 1495)	0.19
**GA (w+d)**	27 + 4 (24 + 3; 34 + 0)	27 + 6 (23 + 4; 29 + 5)	28 + 6 (23 + 1; 33 + 0)	0.43
**APGAR 1**	6 (4; 9)	7 (1; 8)	8 (3; 9)	0.12
**APGAR 5**	8 (5; 10)	9 (5; 10)	9 (1; 10)	0.27
**APGAR 10**	9 (6; 10)	10 (7; 10)	9 (5; 10)	0.59
**Ventilation (d)**	37 (0; 96)	33 (7; 72)	20 (0; 74)	0.18
**Suppl. oxygen**	17/18	18/18	14/18	0.10
**C-section**	16/18	12/18	14/18	0.46
**LOHS (days)**	72 (25; 126)	68.5 (51; 87)	58 (24; 92)	0.04*
**MA (years)**	32 (20; 41)	29 (26; 32)	29 (26; 33)	0.06
**Multiples**	13/18	7/18	8/18	0.71
**UApH**	7.3 (7.17; 7.39)	7.26 (7.09; 7.38)	7.24 (7.0; 7.42)	0.05
**BM feeding**	18/18	0/18	0/18	1.69
**EOS**	5/18	6/18	7/18	0.72
**LOS**	2/18	0/18	1/18	0.38
**I/PVH**	5/18	3/18	4/18	0.78
**RDS**	14/18	12/18	9/18	0.32
**ROP**	6/18	4/18	2/18	0.33
**NEC**	1/18	1/18	0/18	0.61

Data are given as median (min; max) or *n*/total; G= Graz, K= Klagenfurt, L= Leoben, a = years, d = days, w = weeks, m = male, f = female, n = number, g = gram, BW = birth weight, GA = gestational age, APGAR= Appearance Pulse Grimace Activity Respiration Score, suppl. oxygen = supplemental oxygen, LOHS = length of hospital stay, MA = maternal age, BM = breast milk, EOS = early onset sepsis, C-section = cesarean section, LOS = late onset sepsis, I/PVH = intraventricular/periventricular hemorrhage, RDS = respiratory distress syndrome, ROP = retinopathy of prematurity, NEC = necrotizing enterocolitis, UApH = umbilical artery pH. * = differences significant between G and L, and K and L, respectively.

**Table 3:** Time points at the three centers regarding stool sampling of 54 preterm infants <1500 g during the first two weeks of life.

Time Points	Min.	Max.	Mean
t1	1	3	1.56
t2	3	6	3.75
t3	5	8	5.82
t4	7	15	8.04
t5	9	13	10.06
t6	11	15	12.15
t7	13	21	14.50

Data are given as number of days. Min. = minimum, M. = maximum. Stool sampling was planned every second day beginning from the first stool of the preterm infant.
